# The Impact of Baseline Serum C-Reactive Protein and C-Reactive Protein Kinetics on the Prognosis of Metastatic Nasopharyngeal Carcinoma Patients Treated with Palliative Chemotherapy

**DOI:** 10.1371/journal.pone.0076958

**Published:** 2013-10-10

**Authors:** Wei-Xiong Xia, Yan-Fang Ye, Xing Lu, Lin Wang, Liang-Ru Ke, Hai-Bo Zhang, Mark D. Roycik, Jing Yang, Jun-Li Shi, Ka-Jia Cao, Xiang Guo, Yan-Qun Xiang

**Affiliations:** 1 Department of Nasopharyngeal Carcinoma, Sun Yat-Sen University Cancer Center, Guangzhou, P. R. China; 2 State Key Laboratory of Oncology in Southern China, Guangzhou, P. R. China; 3 Department of Biostatistics and Epidemiology, School of Public Health, Sun Yat-Sen University, Guangzhou, P. R. China; 4 Department of Chemistry and Biochemistry and Institute of Molecular Biophysics, Florida State University, Tallahassee, Florida, United States of America; IPO, Inst Port Oncology, Portugal

## Abstract

**Background:**

The aim of this study was to determine whether baseline C-reactive protein (CRP) levels and CRP kinetics predict the overall survival in metastatic nasopharyngeal carcinoma (mNPC) patients.

**Methods:**

A total of 116 mNPC patients from January 2006 to July 2011 were retrospectively reviewed. Serum CRP level was measured at baseline and thereafter at the start of each palliative chemotherapy cycle for all patients.

**Results:**

Patients with higher values of baseline CRP (≥ 3.4 mg/L) had significantly worse survival than those with lower baseline CRP values (< 3.4 mg/L). Patients were divided into four groups according to baseline CRP and CRP kinetics: (1) patients whose CRP < 3.4 mg/L and never elevated during treatment; (2) patients whose CRP < 3.4 mg/L and elevated at least one time during treatment; (3) patients whose CRP ≥ 3.4 mg/L and normalized at least one time during treatment; and (4) patients whose CRP ≥ 3.4 mg/L and never normalized during treatment. The patients were further assigned to non-elevated, elevated, normalized, and non-normalized CRP groups. Overall survival rates were significantly different among the four groups, with three-year survival rates of 68%, 41%, 33%, and 0.03% for non-elevated, elevated, normalized, and non-normalized CRP groups respectively. When compared with the non-elevated group, hazard ratios of death were 1.69, 2.57, and 10.34 in the normalized, elevated, and non-normalized groups (*P* < 0.001).

**Conclusions:**

Baseline CRP and CRP kinetics may be useful to predict the prognosis of metastatic NPC patients treated with palliative chemotherapy and facilitate individualized treatment. A prospective study to validate this prognostic model is still needed however.

## Introduction

Nasopharyngeal carcinoma (NPC) is characterized by marked geographic and population differences in incidence and distribution [1,2]. The primary histological type of NPC is the poorly or undifferentiated pathological type (70%–99% depending on geographic area) [3]. Owing to this characteristic histology and the abundant lymphatic network in nasopharynx, NPC exhibits greater regional and distant metastasis than other squamous cell carcinoma of head and neck (SCCHN). Approximately 6% of newly diagnosed NPC patients have distant metastatic disease at the time of presentation [4] and more than 20% will ultimately develop distant metastasis after definitive treatment using chemoradiotherapy [5,6].

Metastatic NPC (mNPC) exhibits a great deal of variability in its clinical presentation and behavior. Its prognosis is generally poor with a median overall survival of 12 to 15 months [7,8]. Current therapies are palliative chemotherapy, aimed towards prolonging survival, controlling symptoms, and maintaining or improving the quality of life. Nevertheless, several reports indicate that for specific subgroups of patients, depending on the site of metastasis and treatment given, overall survival may exceed ten years [9,10]. As patients with mNPC do not behave uniformly, an easily available and effective biomarker to stratify the patients who would potentially be cured with palliative chemotherapy would greatly enhance clinical decision-making.

Chronic inflammation plays an important role in NPC development and progression. As such, several inflammatory factors, neutrophils, lymphocytes, CCL2, and interleukin-8 (IL-8), are associated with the prognosis of NPC patients [11-14]. C-reactive protein (CRP) is an acute phase protein primarily synthesized in hepatocytes in response to changes in proinflammatory mediators such as interleukin-1 (IL-1), interleukin-6 (IL-6) and tumor necrosis factor-α (TNF-α) [15]. CRP is also a strong prognostic predictor in many cancers, including metastatic renal cell carcinoma, advanced pancreatic cancer, gastric cancer, breast cancer, colorectal cancer, inoperative non-small cell lung cancer, prostate cancer, pancreatic cancer, and esophageal cancer [16,17]. Recently we showed that elevated CRP correlates with heightened metastatic risk in patients with primary NPC [18]. However, to our knowledge there is no report about the prognostic value of CRP and CRP kinetics in patients with mNPC. Therefore, the current study was designed to evaluate the significance of baseline serum CRP level and CRP kinetics on survival in patients with mNPC who received palliative chemotherapy. 

## Materials and Methods

### Patients

The study included 116 patients with histologically proven metastatic NPC treated with palliative chemotherapy between January 2006 and July 2011 at Sun Yat-Sen University Cancer Center. Entry criteria for patients consisted of: (1) good performance status (Karnofsky Performance Scores≥80); (2) normal renal, cardiac, and liver function; (3) complete CRP records, including baseline and thereafter at the start of each palliative chemotherapy cycle; (4) at least two cycles of first line palliative chemotherapy; (5) complete follow-up data; and (6) approved informed written consent. The research ethics committee of the Sun Yat-Sen University Cancer Center approved the study. Written informed consent was obtained from all patients and/or from the next of kin and caretakers.

### Treatment

An overwhelming majority (107, 92.4%) of the patients were treated with platinum-based palliative chemotherapy regimens. The most frequently used regimens included: (1) cisplatin (25–30 mg/m^2^ intravenously on days 1–3 every 21 days) plus 5-fluorouracil (500 mg/m^2^ intravenously on days 1–5 every 21 days) (45, 38.8%); (2) docetaxel (60 mg/m^2^ IV over 3 hours with standard premedication on day 1 of a 21-day cycle) plus cisplatin (60 mg/m^2^ IV on day 1 of a 21-day cycle) plus 5-fluorouracil (600 mg/m^2^, continuous IV infusion for 24 hours, on days 1-5 of a 21-day cycle) (31, 26.7%); (3) paclitaxel (175 mg/m^2^ intravenously over 3 h on day 1 every 21 days) plus cisplatin (25–30 mg/m^2^ intravenously on days 1–3 every 21 cycles) (15, 12.9% ); and(4) cisplatin (25-30 mg/m^2^ IV on days 1-3 of a 21-day cycle) plus capecitabine (1000 mg/m^2^ twice a day by mouth on days 1-14 of a 21-day cycle) (7, 6.0% ). Ten patients received anti-EGFR monoclonal antibody therapy (Cetuximab or Nimotuzomab) and two patients received tyrosine kinase inhibitor therapy (Sorafenib).

### Laboratory measurement

CRP and lactate dehydrogenase (LDH) were measured as described previously [18]. The real-time quantitative PCR system was developed for plasma Epstein-Barr virus (EBV) DNA detection toward the Bam HI-W region. The system consisted of the amplification primers W-44F (5′-AGT CTC TGC CTC AGG GCA-3′) and W-119R (5′-ACA GAG GGC CTG TCC ACCG-3′) and the dual-labeled fluorescent probe W-67T (5′-[FAM] CAC TGT CTG TAA AGT CCA GCC TCC [TAMRA]-3′). Measurement of serum CRP was taken at baseline and thereafter at the start of each treatment cycle.

### Statistical Analyses

Statistical analyses were performed using the statistical package SPSS for Windows version 18.0 (SPSS Inc., Chicago, IL). Overall survival (OS) was calculated from the first day of chemotherapy to the date of death or the last follow-up visit and was estimated by the Kaplan-Meier method and compared between groups via the log-rank test. Multivariate Cox regression model was used to determine whether baseline CRP or CRP kinetics status was a significant predictor of OS. The non-parametric Spearman rank correlation coefficient was used as a measure of correlation between baseline CRP and EBVDNA. All analyses were two-sided and the level of significance was *P*<0.05.

## Results

### Patient characteristics and outcome

As shown in Table 1, the median age of diagnosis of mNPC was 45 years(range, 17-72 years) with men comprising 99 (85.3%) of the patients. Among all patients, 41 (35.3%) had lung metastases, 42 (36.2%) had liver metastases, and 68 (58.6%) had bone metastases at diagnosis. A total of 61 (52.6%) patients had distant metastasis at presentation. During the follow up period with a median of 27 months (range, 4-71 months), 68 (58.6%) of the 116 patients died of metastatic disease. The one-, two- and three-year overall survival rates for the entire patient cohort were 64%, 45%, and 36% respectively.

**Table 1:** Characteristics of patients with metastatic nasopharyngeal carcinoma.

Characteristic	*N* (%)	High baseline CRP (%)	*r*-Value	*P*-value
Age (y)				
≥45	52 (44.8)	32 (61.5)	-0.073	0.436
<45	64 (55.2)	30 (46.9)		
Gender				
Male	99 (85.3)	56 (56.6)	-0.138	0.139
Female	17 (14.7)	6 (35.3)		
Metastasis at presentation				
Present	61 (52.6)	30 (49.2)	-0.125	0.183
Absent	55 (47.4)	32 (58.2)		
Histology, WHO type				
II	5	4	0.113	0.227
III	111	58		
Number of involved sites				
One	69 (59.5)	36 (52.2)	0.177	0.057
Two or more	47 (40.5)	26 (55.3)		
Liver metastasis				
Present	42 (36.2)	26 (61.9)	0.082	0.379
Absent	74(63.8)	36 (48.6)		
Lung metastasis				
Present	41 (35.3)	21 (51.2)	0.040	0.667
Absent	75 (64.7)	41 (54.7)		
Bone metastasis				
Present	68 (58.6)	37 (53.6)	0.005	0.958
Absent	48 (41.4)	25 (53.2)		
Anemia				
Present	32 (27.6)	22 (68.8)	0.278	0.003
Absent	84(72.4)	40 (47.6)		
LDH				
≥192	57	41 (71.9)	0.379	<0.0001
<192	59	21 (35.6)		
EBV-DNA				
≥62800	53	38 (71.7)	0.352	0.0001
<62800	63	24 (38.1)		

Abbreviation: CRP = C-reactive protein; LDH = lactate dehydrogenase; EBV-DNA = Epstein-Barr virus DNA.

The cutoff points of the CRP, LDH, and EBV-DNA according to OS were determined by ROC curve analysis.

### C-reactive protein and C-reactive protein kinetics

The median baseline CRP level was 4.04 mg/L (range, 0.18-130.67 mg/L). Median and mean numbers of CRP measurements were 5 times (range, 2-13) and 5.4 times respectively. The cutoff point of baseline CRP was set at 3.4 mg/L, with the highest value of “sensitive + specificity” in the receiver operating characteristics analysis using overall death as an end point.

**Figure 1:**
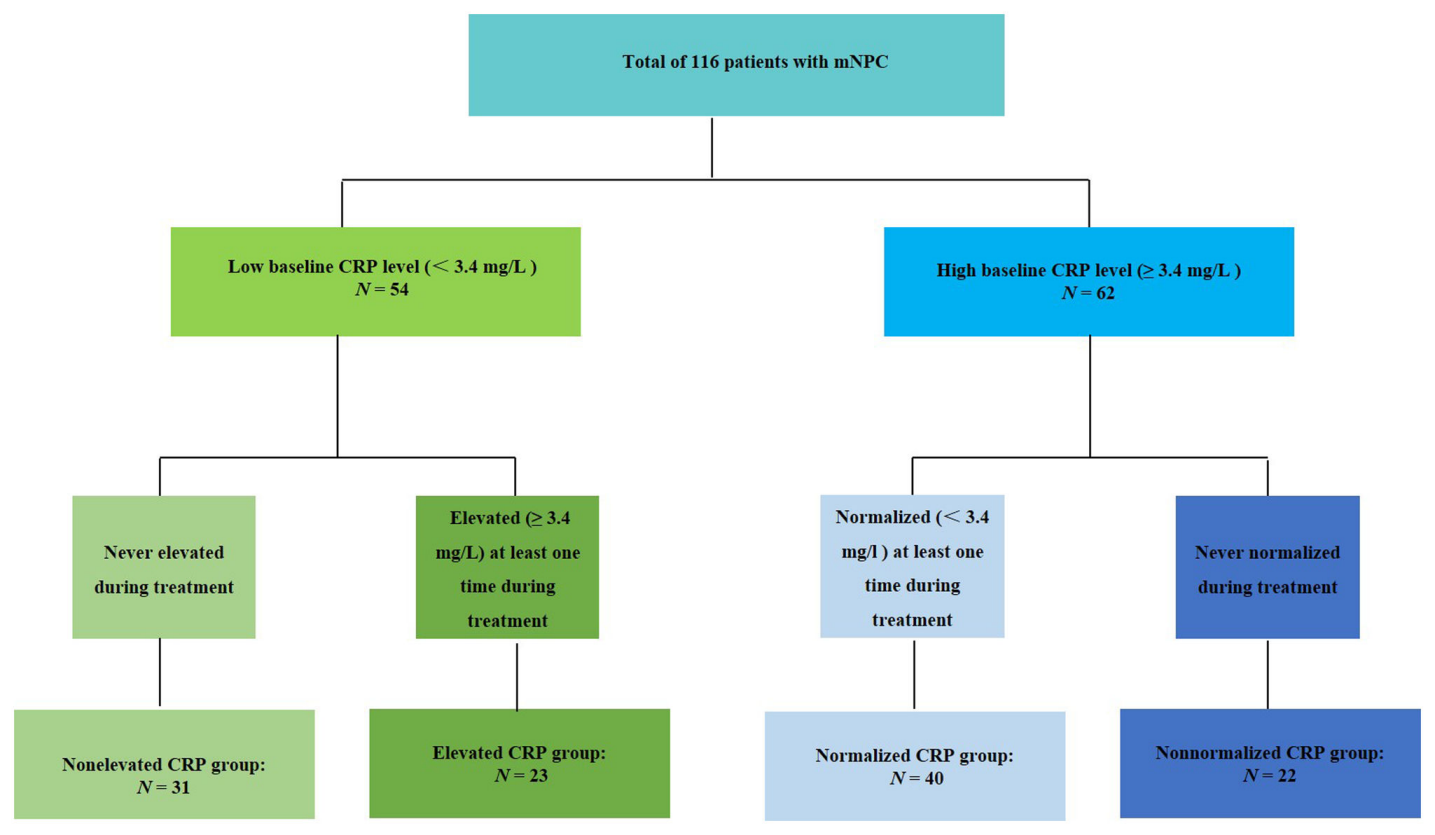
Flow chart of the different patients group according to C-reactive protein (CRP) kinetics.

Patients were divided into four groups according to baseline CRP and CRP kinetics: (1) patients whose CRP < 3.4 mg/L and never elevated during treatment; (2) patients whose CRP < 3.4 mg/L and elevated (CRP ≥ 3.4 mg/L) at least one time during treatment; (3) patients whose CRP ≥ 3.4 mg/L and normalized (CRP <3.4) at least one time during treatment; and (4) patients whose CRP ≥ 3.4 mg/L and never normalized during treatment were assigned to non-elevated, elevated, normalized, and non-normalized CRP groups (Figure 1).

A total of 62 (53.5%) of the 116 patients were with high baseline CRP levels (CRP ≥ 3.4 mg/L) at diagnosis of mNPC. During the treatment, CRP levels in 40 (64.5%) of the 62 patients normalized at least once (normalized CRP group), whereas 22 patients remained elevated during the treatment period (non-normalized group). The remaining 54 patients were with low baseline CRP (CRP < 3.4 mg/L). In 31 (57.4%) of these patients (non-elevated CRP group) CRP levels were not elevated during the treatment, whereas CRP levels were elevated at least one time in the other 23 (42.6%) patients (elevated CRP group).

### Association of baseline C-reactive protein with clinicopathologic characteristics

**Figure 2:**
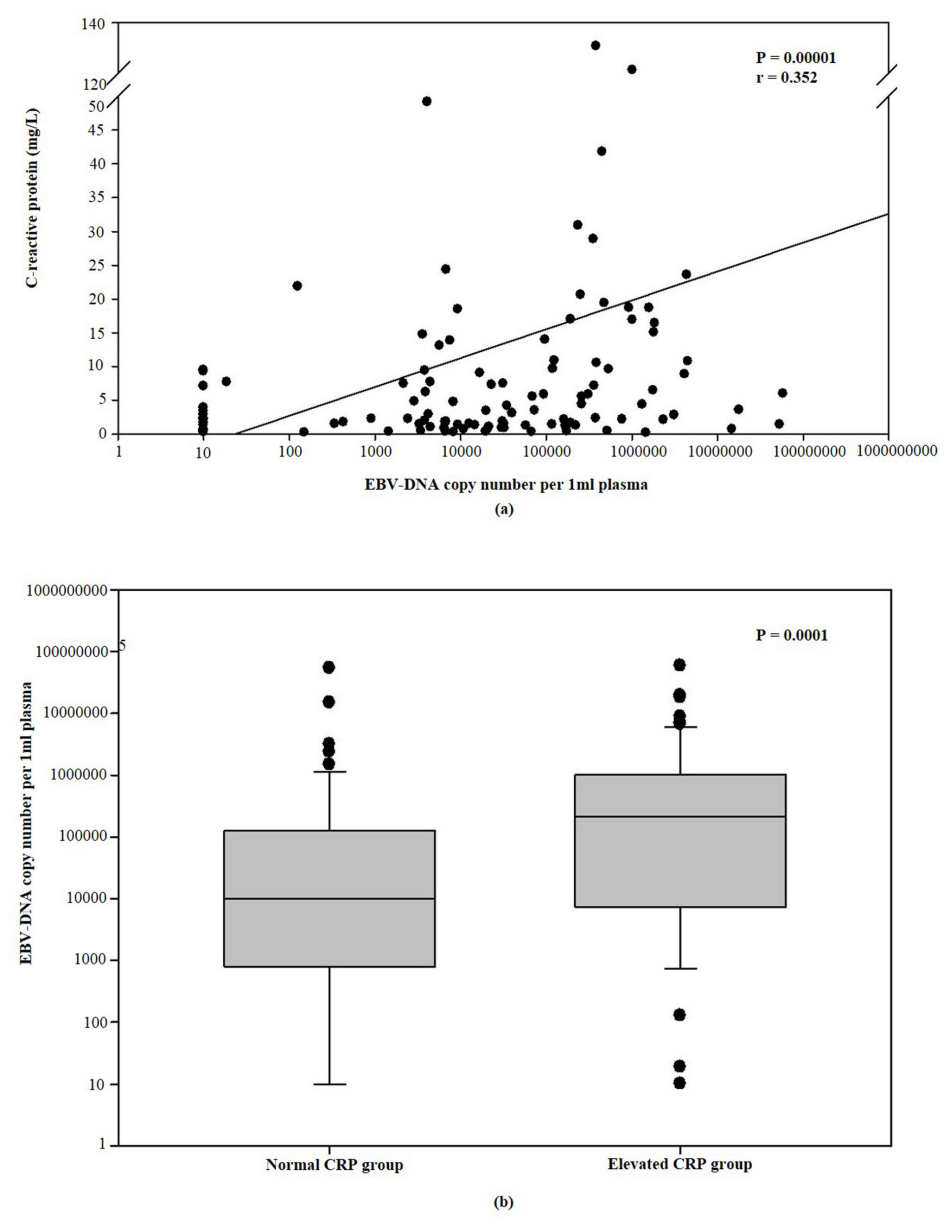
The relationship between the baseline serum C-reactive protein and plasma EBV-DNA copy. (A) Correlation between baseline serum C-reactive protein and plasma EBV-DNA copy (*P* < 0.0001, r = 0.352). (B)The plasma EBV-DNA copy of NPC patients with higher baseline CRP levels was significantly higher than those with lower baseline CRP levels (*P* = 0.0001).

Sex, age, metastasis at presentation, and metastasis sites (i.e., bone, liver, lung), did not influence baseline CRP levels. The baseline CRP levels were significantly associated with anemia (*P* = 0.003, r = 0.278), LDH (*P* < 0.0001, r = 0.379), and plasma EBVDNA copy level (*P* = 0.0001, r = 0.352). CRP levels were marginally associated with more than one metastatic site (*P* = 0.057, r = 0.177). As shown in Figure 2, the median EBVDNA copy of the CRP-higher subgroup was 215500, which was higher than that for the CRP-lower subgroup of 10120 (*P* = 0.001) (Table 1).

### Prognostic of survival

Analyzed factors included age, sex, metastasis at presentation, metastasis sites (i.e., bone, liver, and lung), number of involved sites, anemia, serum LDH, baseline EBVDNA, baseline CRP level, and CRP kinetics status. Univariate analysis revealed that liver metastasis (*P* = 0.006), metastasis at presentation (*P* = 0.018), two or more metastasis sites (*P* = 0.0001), anemia (*P* < 0.0001), higher baseline LDH level (*P* = 0.0002), higher baseline EBVDNA level (*P* < 0.0001), higher baseline CRP (*P* < 0.0001), and CRP kinetics (elevated CRP, normalized CRP and non-normalized CRP, *P* = 0.0001) were considered adverse factors for overall survival (Table 2). In multivariate analysis, anemia, baseline EBV-DNA copy level, baseline CRP, and CRP kinetic statues were independent prognostic factors (Table 3).

**Table 2:** Univariate analysis of prognostic factors with overall survival.

	Events (%)	2-year OS (95%CI)	Unadjusted HR (95%CI)	*P* value
Age (years)				
<45	32 (61.5)	0.38 (0.24, 0.52)	Reference	0.217
≥45	36 (56.2)	0.50 (0.36, 0.63)	0.74 (0.46, 1.20)	
Sex				
Male	59 (59.6)	0.44 (0.34, 0.54)	Reference	0.839
Female	9 (52.9)	0.24 (0.22, 0.70)	0.93 (0.46, 1.88)	
Bone metastasis				
Absent	28 (58.3)	0.38 (0.22, 0.54)	Reference	0.864
Present	40 (58.8)	0.49 (0.37, 0.61)	0.96 (0.59, 1.56)	
Liver metastasis				
Absent	36 (48.6)	0.55 (0.43, 0.67)	Reference	0.006
Present	32 (76.2)	0.26 (0.12, 0.40)	1.93 (1.19, 3.12)	
Lung metastasis				
Absent	41 (54.7)	0.49 (0.37, 0.61)	Reference	0.220
Present	27(65.9)	0.35 (0.19, 0.51)	1.35 (0.83, 2.20)	
Metastasis at presentation				
Present	29 (47.5)	0.58 (0.44, 0.72)	Reference	0.018
Absent	39 (70.9)	0.30 (0.16, 0.44)	1.77 (1.09, 2.87)	
Number of involved site				
One site	34 (46.6)	0.57 (0.45, 0.69)	Reference	0.0001
Two or more sites	34 (79.1)	0.24 (0.10, 0.38)	2.49 (1.53, 4.03)	
T Classification^*^				
T1	3 (75.0)	-	Reference	0.227
T2	18 (75.0)	0.40 (0.20, 0.60)	1.31 (0.39, 4.47)	
T3	31 (50.8)	0.50 (0.36, 0.64)	0.72 (0.22, 2.36)	
T4	16 (59.3)	0.41 (0.21, 0.61)	0.91 (0.26, 3.13)	
N Classification^*^				
N0	5 (50.0)	0.50 (0.19, 0.81)	Reference	0.441
N1	19 (57.6)	0.56 (0.38, 0.74)	1.23 (0.46, 3.32)	
N2	31 (59.6)	0.40 (0.26, 0.54)	1.52 (0.58, 3.97)	
N3	13 (61.9)	0.33 (0.09, 0.56)	2.02 (0.71, 5.78)	
Anemia				
Absent	39 (46.4)	0.58 (0.46, 0.70)	Reference	< 0.0001
Present	29(90.3)	0.24 (0.0, 0.24)	3.45 (2.10, 5.66)	
CRP, mg/L				
<3.4	23 (42.6)	0.67 (0.53, 0.81)	Reference	< 0.0001
≥3.4	45(72.6)	0.25 (0.13, 0.37)	2.89 (1.74, 4.80)	
LDH, U/L				
<192.0	26 (44.1)	0.63 (0.49, 0.77)	Reference	0.0002
≥192.0	42(73.7)	0.24 (0.10, 0.38)	2.42 (1.48, 3.97)	
EBV-DNA				
<62800	24 (38.1)	0.66 (0.54, 0.78)	Reference	< 0.0001
≥62800	44 (83.0)	0.24 (0.08, 0.32)	3.18 (2.16, 5.92)	
CRP kinetics				
Non-elevated	10 (32.7)	0.68 (0.50, 0.86)	Reference	0.0001
Normalized	24 (60.0)	0.66 (0.44, 0.87)	1.69 (0.74, 3.87)	
Elevated	13 (56.5)	0.38 (0.22, 0.54)	2.57 (1.23, 5.39)	
Non-normalized	21 (95.5)	0.03 (0, 0.11)	10.34 (4.63, 23.10)	

Abbreviations: CRP = C-reactive protein; LDH = Lactate dehydrogenase; HR = hazard ratio; CI =confidence interval; The cutoff points of the CRP, LDH, and EBV-DNA according to OS were determined by ROC curve analysis.

* According to American Joint Committee on Cancer (AJCC) 2002 system, and all the stage were initial stage when patients diagnosed with nasopharyngeal carcinoma.

**Table 3:** Multivariate analysis of prognostic factors for patients with metastatic nasopharyngeal carcinoma.

Variable	Category		Model 1		Model 2
		*P*	Exp(B)(95.0% CI)	*P*	Exp(B)(95.0% CI)
	Present				
Liver metastasis	vs.	0.579	1.180 (0.657 to 2.120)	0.396	1.286 (0.719 to 2.303)
	Absent				
	Present				
Metastasis at presentation	vs.	0.985	1.005 (0.570 to 1.774)	0.798	1.080 (0.598 to 1.951)
	Absent				
	One site				
Number of involved site	vs.	0.207	1.500 (0.799 to 2.815)	0.708	1.130 (0.597 to 2.140)
	Two or more sites				
	Present				
Anemia	vs.	0.049	1.790(1.002 to 3.198)	0.018	2.029 (1.130 to 3.641)
	Absent				
	< 192.0				
LDH (U/L)	vs.	0.391	1.294 (0.719 to 2.329)	0.084	1.619 (0.937 to 2.799)
	≥ 192.0				
	< 62800				
BV-DNA	vs.	0.028	1.927 (1.074 to 3.456)	0.029	1.896 (1.068 to 3.367)
	≥ 62800				
	< 3.4				
CRP (mg/L)	vs.	0.042	1.842 (1.023 to 3.315)	-	-
	≥ 3.4				
	Non-elevated				
	vs.				
	Normalized				
CRP kinetics	vs.	-	-	0.000	1.616 (1.241 to 2.106)
	Elevated				
	vs.				
normalized	Non-normalized				

Abbreviations: CRP = C-reactive protein; LDH = Lactate dehydrogenase; HR = hazard ratio; CI = confidence interval;The cutoff points of the CRP, LDH, and EBV-DNA according to OS were determined by ROC curve analysis.

### Baseline C-reactive protein levels and survival

**Figure 3:**
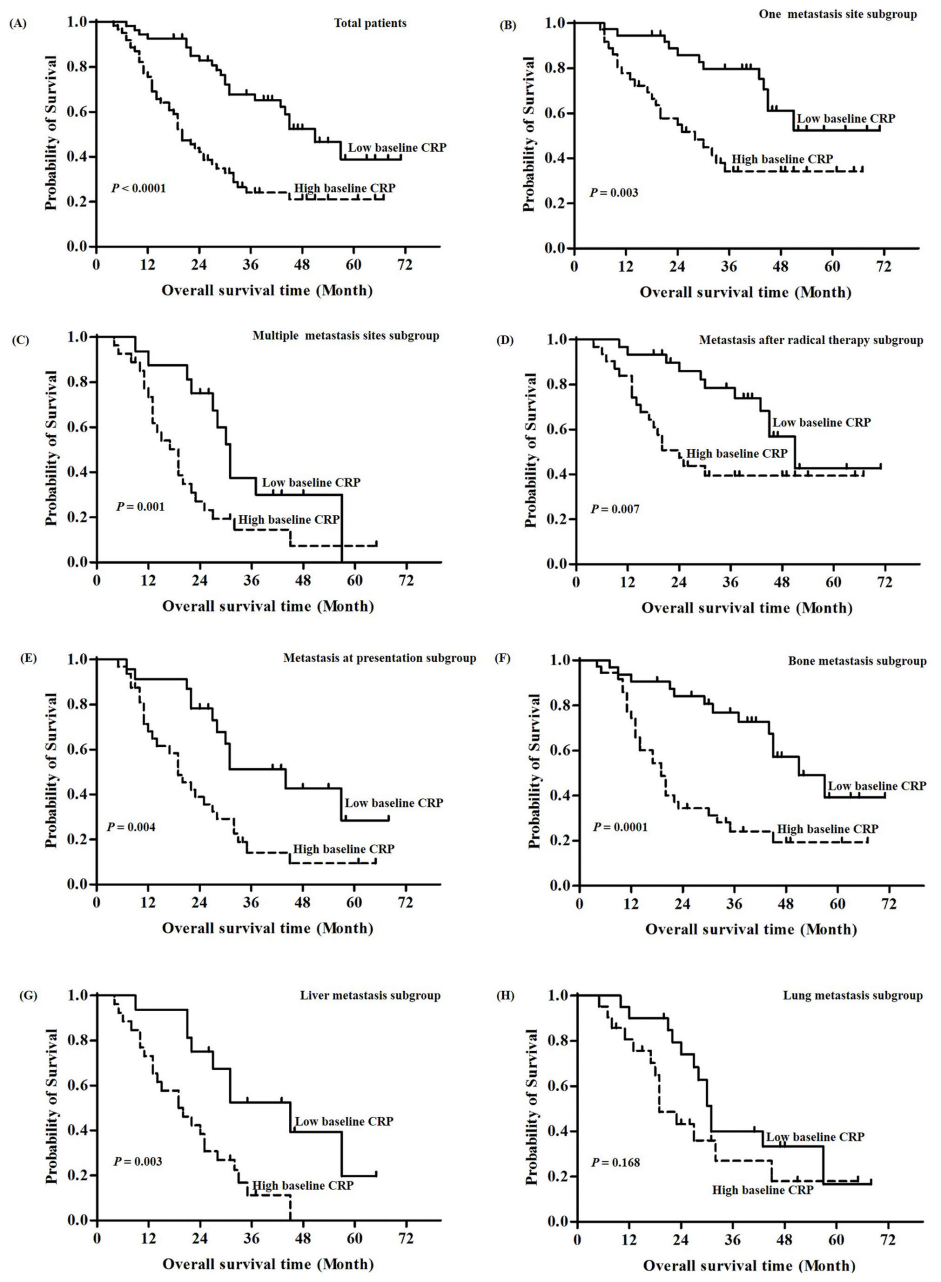
Kaplan-Meier analysis of overall survival according to baseline C-reactive protein (CRP) levels. (A) Overall survival in all patients according to baseline CRP levels (*P* < 0.0001). (B) Kaplan-Meier analysis of overall survival in one metastasis subgroup (*P* = 0.003). (C) Kaplan-Meier analysis of overall survival in multiple metastasis sites subgroup (*P* = 0.001). (D) Kaplan-Meier analysis of overall survival in metastasis after radical therapy subgroup (*P* = 0.007). (E) Kaplan-Meier analysis of overall survival in metastasis at presentation subgroup (*P* = 0.004). (F) Kaplan-Meier analysis of overall survival in bone metastasis subgroup (*P* = 0.0001). (G) Kaplan-Meier analysis of overall survival in liver subgroup (*P* = 0.003). (H) Kaplan-Meier analysis of overall survival in lung subgroup. The log-rank test was used to calculate P-values (*P* = 0.168).

As shown in Figure 3A, patients in the higher CRP level group experienced significantly shorter OS than patients in the lower CRP group (i.e., 3-year OS 25% vs. 67%, *P* < 0.0001) in the global population. Subgroup analysis according to the number of metastases showed that the higher baseline CRP group exhibited worse survival than lower baseline CRP patients in either the one metastatic site subgroup (*P* = 0.003) or the more than one metastatic site subgroup (*P* = 0.001) (Figure 3B & 3C). Also, the higher baseline CRP group had worse survival than lower baseline CRP patients that developed metastasis after radical treatment (*P* = 0.007) and those with metastasis at presentation (*P* = 0.004) (Figure 3D & 3E). Furthermore, stratified analysis according to metastatic organ category showed that the higher baseline CRP group had lower survival than the lower baseline with bone metastases (*P* = 0.0001), as well as those with distant liver metastases (*P* = 0.003). However, for patients with lung metastases both groups showed similar survival (*P* = 0.168) (Figure 3F–3H).

### C-reactive protein kinetics and survival

**Figure 4:**
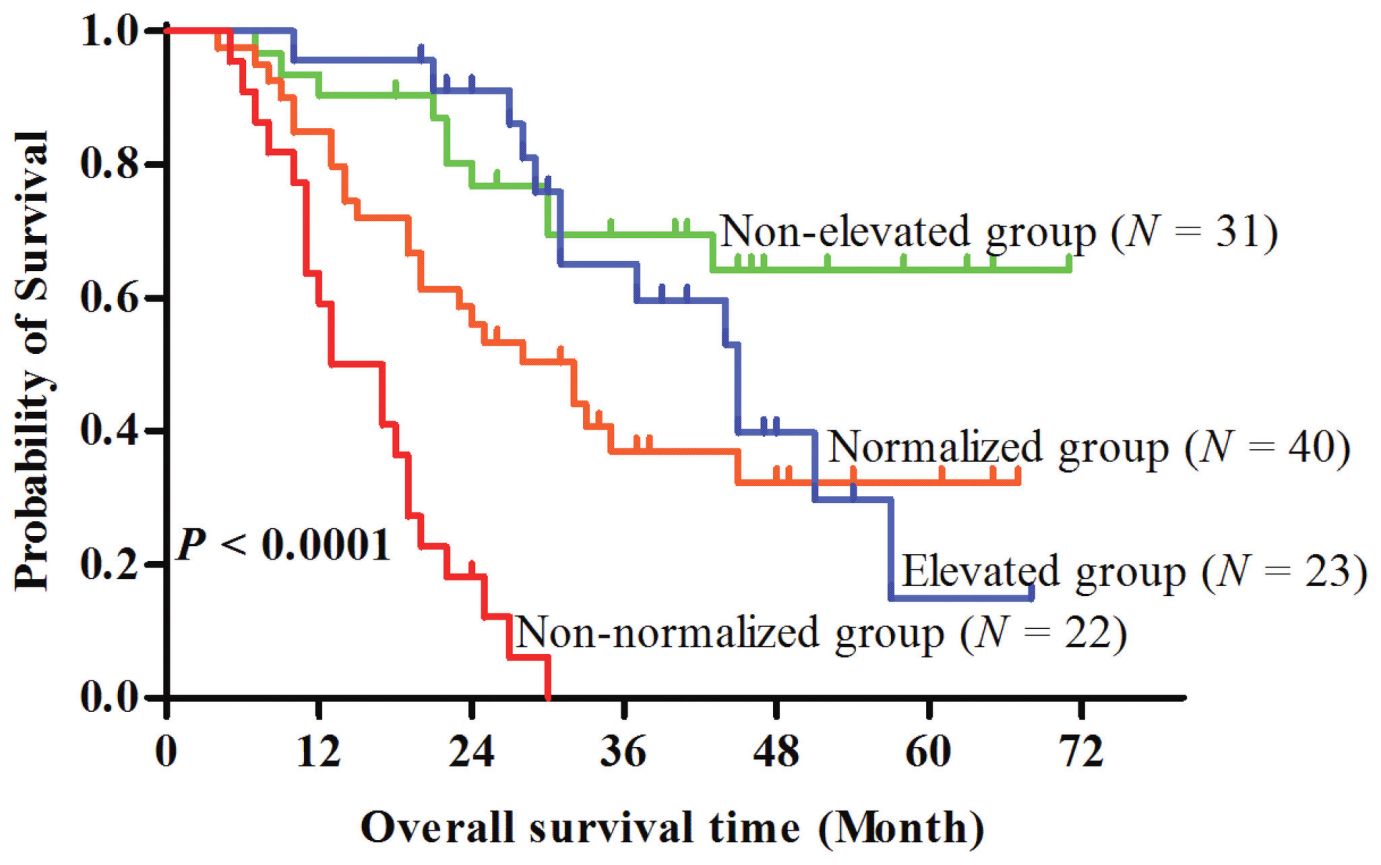
Kaplan-Meier analysis of the overall survival regarding CRP kinetics. Kaplan-Meier analysis of overall survival of non-elevated (CRP < 3.4 mg/L and never elevated during treatment), elevated (CRP < 3.4 mg/L and elevated at least one time during treatment), normalized (CRP ≥ 3.4 mg/L and normalized at least one time during treatment) and non-normalized (CRP ≥ 3.4 mg/L and never normalized during treatment) groups patients with metastasis nasopharyngeal carcinoma (*P* < 0.0001).

During the follow up, 10 (32.7%) of the 31 patients in the non-elevated CRP group, 24 (60.0%) of the 40 patients in the normalized CRP group, 13 (56.5%) of the 23 in the elevated CRP group, and 21 (95.5%) of the 22 patients in the non-normalized CRP group died. Median OS was 60 months in the non-elevated CRP group, 43.5 months in the elevated CRP group, 30.0 months in the normalized CRP group, and 15.6 months in the non-normalized CRP group. There was a significant difference in OS among the four groups (*P* < 0.001) (Figure 4), with three-year OS rates of 68%, 41%, 33%, and 0.03% for the non-elevated, elevated, normalized, and non-normalized CRP groups respectively. When compared with the non-elevated group, the hazard ratios of death were 1.69, 2.57, and 10.34 in the normalized, elevated, and non-normalized groups respectively (*P*<0.001).

## Discussion

In the present study, we demonstrate that CRP helps predict the prognosis for patients with mNPC. Furthermore, our results indicate that combining baseline CRP with CRP kinetics may enhance prognostic prediction of patients with mNPC than baseline CRP level alone. To the best of our knowledge, this is the first report to reveal the prognostic significance of the baseline CRP level and CRP kinetics of patients with mNPC. 

We also report that the presence of an elevated baseline CRP level was significantly associated with elevated baseline of EBVDNA. Previous studies establish a strong association between with Epstein-Barr virus (EBV) infection and NPC [19,20]. Plasma EBVDNA also has been identified for patients in the non-metastatic and metastatic setting as a prognostic predictor [21,22]. The capacity of EBV to induce or affect the expression of cytokines has been studied intensively in EBV-immortalized lymphoblastic cell lines [23-25]. The liberation of multiple proinflammatory cytokines, including IL-1, IL-6, and TNF-α from the tumor microenvironment results in the induction of CRP synthesis from the liver [15]. This suggests that elevated CRP levels might be due, to a certain extent, to the subsequent cytokine stimulation of EBV infection. Further studies need to be performed to clarify this mechanism.

It is reported that liver metastasis, metastasis after radical treatment, anemia, higher baseline LDH level, and higher baseline EBVDNA level are negative factors associated with prognosis of mNPC [26-28]. In the present study, liver metastasis, metastasis after radical treatment, anemia, higher baseline LDH level, and higher baseline EBV-DNA level also were prognostic factors in univariate analysis. However, when these factors were enrolled in multivariate analysis, only CRP, EBVDNA, and anemia were independent factors. Moreover, when stratified to subgroup analysis, baseline CRP showed a particularly strong effect in the subgroup of patients with liver metastasis and metastasis after radical treatment.

 A recent growing body of evidence indicates the prognostic importance of biomarker kinetics in various solid cancers [29-33]. For example, prostate-speciﬁc antigen (PSA) kinetics is signiﬁcantly associated with increased risk of all-cause-mortality among patients with recurring prostate cancer [33]. The serum carcinoembryonic antigen (CEA) kinetic has also been established and is an accurate, simple, and noninvasive method to identify the disease progression in patients with unresectable metastasis of colorectal cancer [30]. The tumor marker CA-125 kinetic is now widely accepted to be an accurate predictive and prognostic factor in CA-125-positive ovarian cancers [29]. CRP kinetics is also a strong prognostic factor in patients with advanced solid tumors, including metastatic renal cell cancer and advanced urothelial carcinoma [31,32]. Consistent with previous studies, the current data demonstrates that CRP kinetics is an independent and significant prognostic indicator in patients with mNPC. Interestingly, the prognosis of patients with CRP elevation during the palliative treatment is worse than those patients without CRP elevation; the prognosis of patients with normalization during the palliative treatment is better than those patients without CRP normalization. These results indicate that serial measurements of CRP may be convenient and useful to estimate the true status of tumor and assess the efficacy of therapeutic intervention in patients with mNPC.

The biological basis for the correlation between the dynamic changes of CRP and disease risk and outcome are not completely understood. We estimate that dynamic changes of CRP may reflect fluctuations of inflammation and dynamic changes of other cytokines, especially IL-6. Also, it is reported that CRP is associated with the nutrition status and development of cachexia [34]. Progressive involuntary weight loss, especially of lean tissue, is common in patients with advanced cancer and has been recognized to develop cachexia and increase morbidity and mortality. It has been reported that an elevated resting energy expenditure in patients with various advanced cancers, such as pancreatic cancer and lung cancer, is associated with the presence of a systemic inflammatory response, as evidenced by an elevated CRP concentration [35-37]. Furthermore, elevated serum CRP levels are associated with higher tumor burden and advanced tumor stages, which will, to certain extent, be related to the dynamic changes of CRP. Recently, CRP was also found to be a monitor of chemotherapy response in advanced non-small cell lung cancer [38]. The same results were also reported in the Glasgow Prognostic Score (GPS) system, a prognosis model based on CRP and albumin, which can effectively predict the treatment outcome of various cancers [39,40].

Our study does have limitations. First, a sample size of 116 is modest for an analysis of prognostic markers in this patient population with a larger, multicenter design needed for further study. Second, given the difficulty in evaluating bone metastasis, the relationship between CRP and chemotherapy response is still unknown. We plan to start a prospective, multicenter study to verify the prognostic value of baseline CRP and CRP kinetics in the near future.

In conclusion, the baseline CRP and CRP kinetics may be useful to predict the prognosis of mNPC patients treated with palliative chemotherapy and facilitate individualized treatment. A prospective study to validate this prognostic model is needed.
